# Reply to the Comment by Littlechild and Isupov

**DOI:** 10.3390/microorganisms5030055

**Published:** 2017-09-06

**Authors:** Andrew Willetts, David Kelly

**Affiliations:** 1 College of Life and Environmental Sciences, University of Exeter, Exeter EX4 4QG, UK; 2 School of Chemistry, Cardiff University, Cardiff CF10 3AT, UK

I thank Drs. Littlechild and Isupov for their recent comments, which are considered below. Before addressing these specifically, their correspondence raises two more general issues which require initial clarification. 

## General Issues

Firstly, all the observations made in doi:10.3390/microorganisms4040038 [1] about the “model/structural model” refer exclusively to material presented in Sections 3.5–3.7 in [2]—none of the comments refer to the experimentally determined crystalline structures of 3,6-DKCMO (PDB code: 4UMW) presented in Sections 3.2 and 3.3. This caveat is also relevant to observations made elsewhere [3,4]. As evidenced previously [5], misconceptions arise when that important distinction is not appreciated and respected, either by default or design. 

Secondly, Drs. Littlechild and Isupov’s assertion that “In the paper [1] the lead author Willetts has described work carried out by himself and Dr David Kelly prior to 2008” is incorrect. As evidenced below (*vide supra*), correct facts and timelines are both important matters: therefore, to reiterate the statement made in the “Author Contribution” entry of [1], while Dr. David Kelly’s major contribution was to the conception and overall design of the research programme prior to his sudden unexpected death in 2008, most of the subsequent stages (gathering and analysing data, and writing the paper) were conducted exclusively thereafter by Andrew Willetts.

## Specific Issues

Important to gaining a full comprehension of both the issues raised by Drs. Littlechild and Isupov’s Comment and the prior observations made [1,3,4] regarding Isupov et al.’s modelling studies is the fact that the oxidation of reduced flavins by oxygen-dependent enzymes, including Type I and II Baeyer-Villiger monooxygenases (BVMOs: [6]), has been extensively studied for several decades. Consequently, both structural and functional aspects of a significant number of representative examples, including various flavin-diffusible two-component monooxygenases (fd-TCMOs: [7]), are very well characterised and understood. 

Both Type I and II BVMOs catalyse the same sequence of key biochemical events involving critical patterns of hydrogen bonding from key active site residues to the reduced flavin N5-H which is crucial for the successive formation and stabilisation of firstly a C4a-hydroperoxyflavin, and subsequently a Criegee intermediate. The relative movement of domains is essential for this series of sequential catalytic steps leading to lactone formation and release [8,9,10,11], and structures typically consist of semi-rigid domains connected via flexible regions or hinges [12,13]. These are the crucial definitive structural and functional features which ensure that all BVMOs (Types I and II) can undertake their unique idiosyncratic role as efficient oxygen-activating and “Criegee-stabilizing” catalysts. Any BVMO-based publication dealing with aspects of either structure and/or function that fails to embrace these highly relevant recent developments is likely to be an anachronism.

Isupov et al.’s study [2] of 3,6-diketocamphane monooxygenase (3,6-DKCMO), an fd-TCMO from camphor-grown *Pseudomonas putida* NCIMB 10007, determines to a high resolution (1.9 Å and low R-values) the three-dimensional structures of both the native apo enzyme and the corresponding oxidised flavin (FMN)-bound complex, which have been deposited with the PDB (code: 4UWM). In strict contrast, however, their modelling proposals detailed in Sections 3.5 and 3.6 of their paper (FMN cofactor modelling and active site plus FNR modelling, respectively) offer no sound biochemical perspective on which amino acid residues promote the formation and stabilisation of the C4a-hydroperoxyflavin and Criegee intermediates essential for catalytic activity, and consequently offer no mechanistic insight on 3,6-DKCMO (*vide infra*, [8,9,10,11,12,13]). The binding of FMN proposed by Isupov et al., which they report as retained by the reduced form of the flavin FNR, is based on the electron density shown in Figure 3 of [2]. Significantly, due to the quality of this data, it is not possible to accurately assign the orientation of the isoalloxazine ring of FMN from these crystallographic data, and consequently the location of N5 and the heterocyclic moiety of this tricyclic ring, which both serve as key determinants of functional activity, is ill-defined. Figures 4a and 5 of their 2015 paper therefore have little experimental basis.

It is likely that a combination of structural and biochemical factors may be contributing to this stark dichotomy between high resolution and low functional definition.

### *Structural* *Considerations: How Valid Are Isupov et al.’s Solved Crystal Structures?*

Here, the relative timing of key relevant events is an important issue in two different contexts.

Firstly, it was only in 2013 that 3,6-DKCMO was characterised correctly as an fd-TCMO [14]: like all such enzymes, FMN has no direct involvement with the sequence of biochemical events catalysed by the oxygenating moiety. However, at the time the structure of native apo 3,6-DKCMO was solved (2008: [15]), the enzyme was still, as it had been for over 40 years, mistakenly believed to be a flavoprotein using bound FMN as a coenzyme which is reduced in situ in the active site by NADH [16]. This may explain why Isupov et al. chose this biochemically inert form of the flavin cofactor (*vide supra*) in developing their structural studies. The structure of the FMN complex was solved by MR with the refined structure of native apo 3,6-DKCMO, which in turn was solved by dependency on MR using a synthetic α2 dimer of the luciferase from *Vibrio harveyi* [15], derived from Fisher et al.’s 1995 2.4 Å resolution of the crystal structure of the native apo α/β heterodimeric form of this bacterial enzyme (PDB code, 1luc: [17]). 

Here, for a second time, as in the case of the mistaken identity of the true nature of 3,6-DKCMO (*vide infra*), relative timing is a highly relevant issue with respect to Isupov et al.’s deployment of the synthetic α2 dimer [15] to aid in solving the structure of native apo 3,6-DKCMO. This is because Fisher et al.’s 22-year old study [17], from which the synthetic α2 dimer was derived, predates proper appreciation of the relevance of structural allostery in influencing the conformation, and hence the functional activity, of ligand-regulated heteromultimeric proteins (recent review: [18]). Thus whereas 3,6-DKCMO is homodimeric, *V. harveyi* luciferase is an α/β heterodimer in which the β subunit, although not catalytically active, is known to have a profound allosteric effect on the 3D shape and hence the catalytic activity (greater than five-fold) of the α subunit resulting from a structural allosteric pathway triggered by the binding of one or more effector ligands. In this respect, the more recent study of this luciferase by Campbell et al. (PDB code, 3FGC: [19]) is doubly important because not only did it take into account the by-then confirmation of the luciferase as an fd-TCMO [20], but it also recognised and partially characterised this β subunit–triggered allosteric effect. Specifically, it highlighted the importance of a mobile loop adjacent to the active site in promoting key aspects of intersubunit communication, a structural feature that was crystallographically disordered in Fisher et al.’s earlier study. 

Because their study did not reflect the relevant structural allosteric pathway and associated domain movements, the outcomes from Fisher et al.’s 1995 crystallographic study [17] correspond to a catalytically inactive structure. Consequently, Isupov et al.’s dependence on a synthetic α2 dimer [15] derived therefrom to help resolve the structure of 3,6-DKCMO will be detrimentally influenced, especially with respect to the functional definition, as would any other similar structural resolution based on this particular synthetic α2 dimer. 

### *Biochemical* *Considerations: Functional Factors Relevant to the Active Site*

There are three principal important biochemical issues that are likely to be influential:

1. In terms of function, the difference between a flavoprotein-type monooxygenase and a flavin-diffusible monooxygenase (*vide infra*) is crucial. In the specific case of 3,6-DKCMO, this is reflected starkly by the fact that relevant kinetics confirm that the respective *Kd* values for FMN and FNR differ by >500% [1]. This indicates that while the reduced form of the flavin is bound very tightly in the active site of the enzyme, FMN exhibits poor affinity (as commented on a number of times by Isupov et al. [2]), implying that any relevant bonding will be weak, and probably largely random, as recorded with a number of other flavin-diffusible TCMOs [7]. These data therefore suggest that the active site modelling proposals presented in [2], which predict that FMN and FNR share a common pattern of H-bonding within the active site of 3,6-DKCMO, are incorrect. Furthermore, their modelling studies with the flavin cofactors place *Phe203* more proximal to the ribityl chain than the benzenoid moiety of the isoalloxazine ring, so it seems unlikely as suggested that “additional favourable hydrophobic interactions with the side chains of *Phe203* and *Ile223*” can alone account for the very large difference (great than five-fold) in binding affinity between the two forms of the flavin cofactor.
2. Both 3,6-DKCMO and *V. harveyi* luciferase have experimentally confirmed opposite facial diastereoselectivity with respect to the alignment of FNR and the divestment of its reducing power within the active sites of the two enzymes [21]. Isupov et al. claim, incorrectly, that 3,6-DKCMO and “all other enzymes of the bacterial luciferase superfamily catalyse their reaction on the (*si*)-side of the ring” [2], and therefore fail to factor this important dichotomy into their modelling studies with the flavin cofactors. If this important biochemical difference had been appreciated, then Figure 5 of [2] might have been interpreted differently.
3. Whereas the isoalloxazine ring of FMN retains a planar conformation when located in the active site of a flavin-dependent protein, the corresponding reduced tricyclic ring of FNR may assume a so-called “butterfly” conformation: the recorded angle of arcing about the N5-N10 axis can range from 1°–38° and is an idiosyncratic characteristic of an individual flavin-dependent protein type. In their own words, “the FNR isoalloxazine ring was modelled with a 20° deviation from planarity, as observed in the structure of…the F_420_-dependent secondary alcohol dehydrogenase (Adh) enzyme” [2]. Their choice of 20° was purely arbitrary (the figure quoted for Adh is actually 28° [22]). Further, the choice of the F_420_ cofactor as a precedent raises significant concerns because when compared to FNR, the tricyclic ring structure of F_420_ is devoid of both the mechanistically crucial N5 atom, and the structurally important pattern of dimethyl substitution on the benzenoid moiety. In commenting on this issue, Drs. Littlechild and Isupov fail to understand the correct use of the word ‘modelled’ in the relevant context in [1], where it is used specifically as the past tense of a verb with the equivalent meanings of “based on”.

Collectively, these various individual structural and biochemical considerations may each contribute to a greater or lesser extent to the striking contrast between the high resolution and low functional definition which characterises Isupov et al.’s studies of 3,6-DKCMO [2]. 

Some or all of these various issues need to be considered in undertaking any attempt [5] to resolve the three-dimensional structure of either of the two isoenzymic forms of 2,5-DKCMO [14], the enantiocomplementary diketocamphane-oxygenating enzyme induced in camphor-grown *Pseudomonas putida* NCIMB 10007. This is especially so if any reliance is placed on the synthetic α2 dimer [15] of the luciferase from *Vibrio harveyi*, as previously emphasised [23].
